# Summation of Precordial R Wave Amplitudes, a Clinical Parameter for Detecting Early TTR Amyloidosis Cardiac Involvement

**DOI:** 10.3390/jcdd9100348

**Published:** 2022-10-11

**Authors:** Yoshitaka Isotani, Eisuke Amiya, Junichi Ishida, Issei Komuro

**Affiliations:** 1 Department of Cardiovascular Medicine, Graduate School of Medicine, University of Tokyo, Hongo 7-3-1, Bunkyo-ku, Tokyo 113-8655, Japan; 2 Department of Therapeutic Strategy for Heart Failure, University of Tokyo, Hongo 7-3-1, Bunkyo-ku, Tokyo 113-8655, Japan

**Keywords:** transthyretin amyloid cardiomyopathy, electrocardiogram, poor R wave progression

## Abstract

There have been several reports on the identification of the stage of transthyretin amyloid cardiomyopathy (ATTR-CM); however, a staging system for ATTR-CM has not yet been established. An 83-year-old woman was referred to our department about ten years ago. Recently, she was diagnosed with ATTR-CM. The electrocardiogram showed characteristic changes that take place over the duration of ATTR-CM progression. Among these, the precordial R amplitudes abruptly decreased before the development of increased ventricular thickness. This case suggested that the decrease in the precordial R wave amplitudes may represent a new diagnostic clue reflecting early myocardial damage due to ATTR-CM.

## 1. Introduction

Several therapeutic agents have been developed for the purpose of transthyretin amyloid cardiomyopathy (ATTR-CM) [1]. As a result, the clinical importance of the accurate diagnosis and evaluation of the stage of cardiac amyloid deposition is growing. The early diagnosis of ATTR-CM is critical, as the prognosis of ATTR-CM rapidly worsens as amyloid deposition continues, accompanied by progressive organ damage. There have been several reports on the identification of the stage of ATTR-CM; however, a staging system for ATTR-CM has not yet been established. Furthermore, the usefulness of each modality for the early detection of ATTR-CM is still being debated.

The present case of ATTR-CM represented characteristic changes in the electrocardiogram (ECG) with the progression of ATTR-CM over a 10-year period.

## 2. Case Presentation

An 83-year-old woman undergoing treatment for hypertension and sleep apnea syndrome was referred to our department in 2014 for shortness of breath on exertion. The ECG results were within the normal limits. Echocardiography showed normal contractility, without an evident increase in ventricular thickness, and cardiac magnetic resonance (CMR) scans showed no significant stenosis in the coronary arteries.

In 2016, her shortness of breath worsened. Echocardiography showed only grade II diastolic dysfunction. However, no other abnormalities were detected that could explain her symptoms. The blood pressure was controlled, and the patient was under observation.

**Figure 1:**
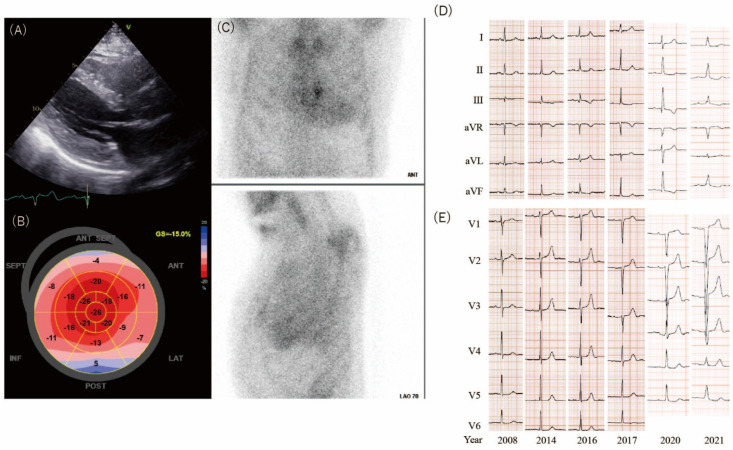
(**A**) Echocardiography in 2020 showing left ventricular hypertrophy (interventricular septum, 11 mm; posterior wall, 11 mm). (**B**) Relative apical sparing by longitudinal speckle tracking strain imaging. (**C**) Technetium hydroxymethylene diphosphonate showing diffuse radiotracer uptake in the myocardium. (**D**) Electrocardiogram change over time in the limb leads. (**E**) Electrocardiogram change over time in the precordial leads.

In 2020, she developed numbness in both hands, which suggested the presence of carpal tunnel syndrome (CTS). At that time, increased ventricular thickness, observed on echocardiography, was evident for the first time (Figure 1A,B). Technetium-99 pyrophosphate imaging demonstrated diffuse, moderate uptake in the myocardium (Figure 1C), and myocardial biopsy revealed ATTR amyloidosis. Genetic testing revealed a diagnosis of wild-type ATTR (ATTRwt). Tafamidis was initiated in December 2020.

**Figure 2:**
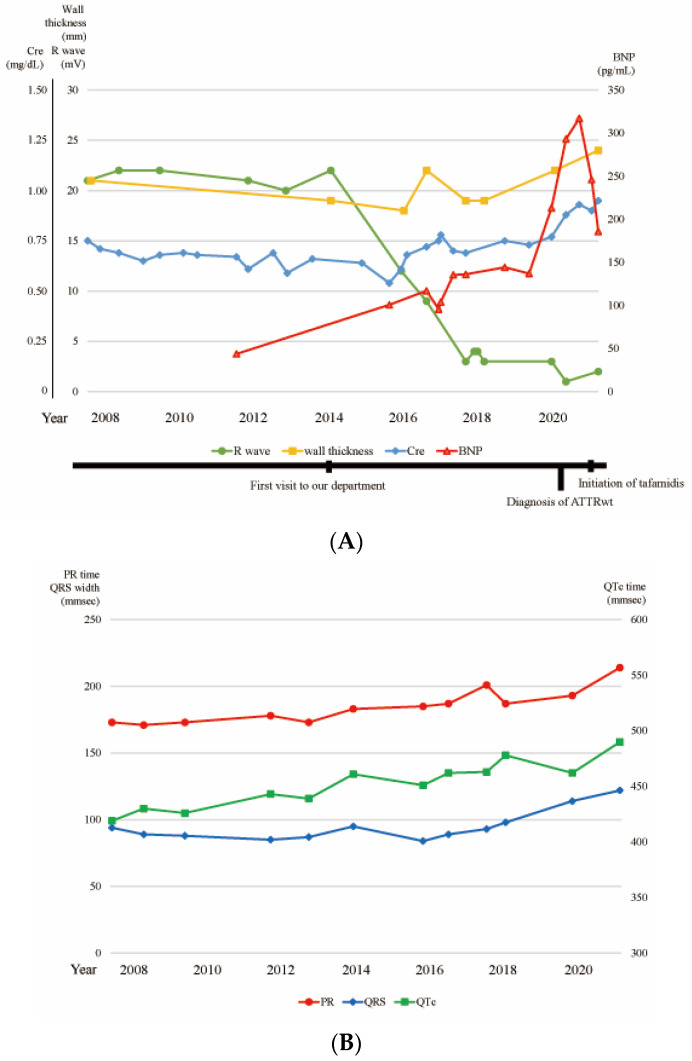
(**A**) Trends in the sums of the R waves in the V1–3 leads on electrocardiography (green line, ECG), the sums of the septal and posterior wall thickness on echocardiography (yellow line, UCG), the level of creatinine (blue line, Cre), and the level of B-type natriuretic peptide (red line, BNP). (**B**) Trends in PR time (red line), QRS width (blue line), and QTc time (green line).

According to the detection of the early signs of ATTR-CM using ECG, her ECGs, which were taken every 1–2 years from 2007 onwards, were analyzed retrospectively. No abnormalities were evident on ECG until 2014. Then, the R waves of leads V1–3 abruptly decreased in 2016 (Figure 1D,E), which preceded the detection of increased ventricular thickness by echocardiography in 2020. Figure 2A shows the sum of the R wave amplitude in leads V1–3 and the sum of the septal wall thickness and posterior wall thickness on echocardiography. The sum of the R wave amplitude reduced from 22 mm in 2014 to 11.5 mm in 2016. In contrast, the sum of the wall thickness gradually increased from 2020 onwards. The brain natriuretic peptide (BNP) and creatinine levels started to increase around the time when the ECG changes appeared (Figure 2A).

In contrast, there were no overt changes in the findings of the low potentials, PR duration, or QTc time before the detection of increased ventricular thickness. The increase in the QRS width became evident after 2020.

Despite the initiation of tafamidis, recent ECG findings showed that the PR duration, QTc time, and QRS duration were all increased (Figure 2B).

The ECG parameters were calculated by a visual assessment (R wave summation) and partly by computer-aided assessment (PR interval, QRS interval, and QTc duration). The LV thickness was measured using a long-axis view of a still image to detect the end-diastole with 2D tracing.

**Figure 3:**
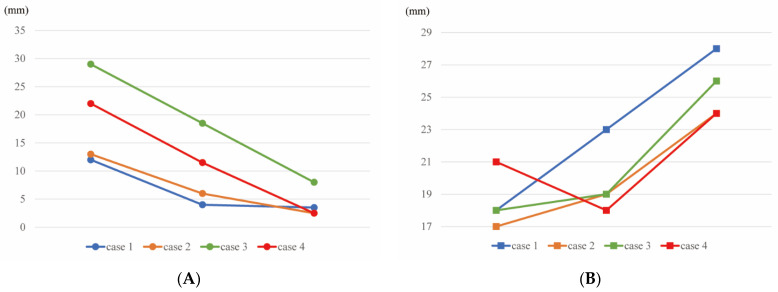
Trends in the summation of the R waves in the V1–3 leads on electrocardiography and the sums of the septal and posterior wall thickness on echocardiography for four ATTR-CM cases (additional three cases and the main case). (**A**) The sum of R amplitudes of V1, V2 and V3 on electrocardiograms. (**B**) The sums of the septal and posterior wall thickness on echocardiography. (**A**,**B**) The 1st point: baseline; 2nd point: time when the ECG change appeared; 3rd point: time when increased ventricular thickness appeared clearly on echocardiography in the 4 cases of ATTR-CM. Panel (**A**) shows a significant difference between the 1st and 2nd points; however, panel (**B**) does not. Both panels show a significant difference between the 1st and 3rd points.

In addition, we analyzed other cases in order to verify relationship between the summation of the amplitudes of the R waves in the V1–3 leads and increased ventricular thickness in ATTR-CM over time. We screened cases taken from our ATTR-CM cohort, which consisted of 32 cases with ATTRwt and 15 cases with a variant type of ATTR. From these, we extracted seven cases, for which a series of ECGs were recorded before the onset of increased ventricular thickness. Among the seven cases, three cases were excluded because two patients showed poor R progression after the first visit, and one patient had complete right bundle branch block. After excluding these cases, four cases (including the main case) remained, as described in Figure 3A,B. Figure 3A,B shows the gradual decrease in the summation of the amplitudes of the R waves during the progression of ATTR-CM. There was a significant decrease in the summation of the R wave amplitudes before increased ventricular thickness was detected by echocardiography.

## 3. Discussion

ATTR-CM, which was previously considered as a rare condition, is increasingly recognized as a cause of heart failure in the elderly [1]. There is a report on postmortem examinations which demonstrated that cardiac amyloidosis was frequently observed in autopsied hearts from patients > 75 years old, especially in patients with heart failure, left ventricular hypertrophy (LVH) and atrial fibrillation [2]. Recently, several reports demonstrated that in about 10–20% of patients with heart failure and LVH, ATTR-CM was identified [3,4]. However, the diagnosis of ATTR-CM is generally established at a late stage of the disease because of the difficulty involved in its diagnosis [5]. Therefore, there might be many cases where treatment is insufficient due to delays in the definite diagnosis of ATTR-CM.

In addition, the natural history of cardiac dysfunction is not well understood in patients with ATTR. Technetium-labeled cardiac scintigraphy and CMR have proven to be useful for the diagnosis of ATTR-CM in patients with a high pre-test likelihood, but its usefulness is limited to a screening method, and the validity of frequent evaluation using these modalities to assess the grade of TTR deposition has not yet been demonstrated [6,7,8]. There are some reports that assert that the severity and prognosis of ATTR-CM can be predicted using a combination of several biomarkers [9,10], but the usefulness of the detailed monitoring of myocardial lesions is ill-defined and insufficient. Furthermore, in order to determine the appropriate timing for starting specific treatments, it is most important to understand the progress of amyloid deposition in the context of ATTR-CM in detail.

For the effective monitoring of the progression of ATTR-CM, ECG might be a possible candidate for disease stage monitoring because of its simplicity. Typical ECG changes seen in ATTR include a low voltage, pseudo-infarct pattern, poor R wave progression (PRWP), atrioventricular block and atrial arrhythmias [11,12].

The definition of PRWP is an R wave amplitude of 3 mm or less in the precordial lead in V3, and an R wave amplitude in the lead in V2 that is equal to or smaller than the R wave amplitude in V3 [13]. Although PRWP is not a specific aspect of TTR amyloidosis, Nicole et al. reported that PRWP was observed in 60–70% of cases, which reflected the most frequent pattern of ECG changes in ATTR-CM [14].

Regarding the findings of a low voltage, the prevalence was reported to be dependent on the definition. They also reported that low voltages based on limb leads lower than 0.5 mV and precordial leads lower than 1.0 mV were infrequent in ATTR-CM patients (6–38%). Low voltages based on the Sokolow criteria lower than 1.5 mV were more common (53–58%). Other ECG findings related to ATTR-CM include the increase in the PR interval, QRS width, PR interval, and QTc, which are, however, less frequent than PRWP [14]. Light-chain amyloidosis has similar but different characteristics on ECG, but in this study, we focused only the characteristics ATTR-CM on ECG [15,16,17]. In addition, there are some reports that have demonstrated that the ECG pattern might predict the clinical course of ATTR-CM [14]. However, PAWP, which was investigated in this study, was not a risk factor for a poor prognosis of ATTR-CM; however, a change in PAWP might correspond to the progression of ATTR-CM.

According to the change in the ECGs observed in this case over time, the changes in the PR interval and QTc time were too slight to notice. The increase in the QRS was observed with the appearance of obvious cardiac hypertrophy. Only PRWP was identified as an abnormal change prior to the detection of cardiac hypertrophy, suggesting that PRWP could detect the earliest myocardial change in the TTR deposition. Indeed, we could verify the usefulness of the summation of the amplitudes of the R waves in the V1–3 leads based on the detection of the progression of ATTR-CM in the other cases (2 cases of ATTRwt and 2 cases of variant-type of ATTR). Figure 3A,B shows the gradual decrease in the summation of the amplitudes of the R waves during the progression of ATTR-CM. There was a significant decrease in the summation of the R wave amplitudes before increased ventricular thickness was detected by echocardiography.

While there are few reports that explain the relationship between the ECG changes and the stage of ATTR amyloidosis, it has been proposed that the ECG changes in Fabry disease reflect the stage of the disease to some extent. In Fabry disease, short P waves and specific T wave patterns are confirmed before the detection of increased ventricular thickness on echocardiography, and the ECG reveals characteristic changes as the disease progresses [18]. As in the case of Fabry disease, it is reasonable to expect that there a specific pattern of ECG changes will emerge as the disease progresses.

The mechanism for the appearance of a specific ECG pattern before the development of increased ventricular thickness may be related to the multistep process of amyloid deposition. A recent report suggested that organ damage occurs at the stage of nonfibrillar TTR before amyloid deposition was observed in the organ [19]. In ATTR, myocardial damage is expected to precede the development of increased ventricular thickness due to amyloid or nonfibrillar TTR deposition, which might be reflected in the ECG changes.

Our case suggests that the decrease in the R wave amplitudes of the V1–3 leads may appear earlier than the echocardiographic findings of cardiac hypertrophy. Among the ECG changes characteristic of ATTR-CM, the summation of the amplitudes of the V1–3 R waves is the most obvious change in the early stage of ATTR-CM. The monitoring of ECG changes is easy and cost-effective and is likely to be useful for the early detection of myocardial damage in ATTR patients, especially in patients with asymptomatic TTR variants or in patients with TTR detected in other organs. Further research is needed in order to investigate the relationships between the various findings of different modalities.

There is one limitation of this case series. The parameters of the ECG and increased ventricular thickness on echocardiography are generally subject to intra- and inter-observer viabilities. In order to solve this problem to the greatest extent possible, the parameters were plotted at numerous time points, which might have lessened the effects of variations in the individual parameters measured (Supplementary Table).

In conclusion, the decrease in the R wave amplitudes of the V1–3 leads may be a new diagnostic clue reflecting early myocardial damage due to ATTR-CM, and this finding could enable the concise follow-up of gradual changes in the TTR deposition, leading to a more appropriate therapeutic strategy.

## Data Availability

Not applicable.

## Supplementary Materials

The supporting information can be downloaded at: https://www.mdpi.com/article/10.3390/jcdd9100348/s1. Supplemental Table: Case 1 (variant ATTR-CM, 31-year-old, male), Case 2 (variant ATTR-CM, 23-year-old, female), Case 3 (ATTR-CMwt, 70-year-old, male).
