# Supplementary material for: Summation of Precordial R Wave Amplitudes, a Clinical Parameter for Detecting Early TTR Amyloidosis Cardiac Involvement
Source: J Cardiovasc Dev Dis. 2022 Oct 11;9(10):348. doi: 10.3390/jcdd9100348 (PMC9604937; doi:10.3390/jcdd9100348)
Supplement: Supplementary file 1 [file jcdd-09-00348-s001.zip › jcdd-1914252-supplementary.pdf]

Supplemental table

Supplementary table shows the details of 3 cases described in Figure 3A, B. 2 cases are variant type of transthyretin amyloid cardiomyopathy (variant ATTR-CM), and 1 case is wild type transthyretin amyloid cardiomyopathy (ATTR-CMwt). The age of each patient is shown in year X. Electrocardiography parameters mean R wave amplitudes in V1-3 leads. Year X is the year at the time of initial examination (shown as 1st point in Figure 3A, B). The second findings are the parameters at which electrocardiographic changes appeared before cardiac hypertrophy became apparent (shown as 2nd point in Figure 3A, B). The third findings are the parameters at which cardiac hypertrophy measured by echocardiography became evident (shown as 3rd point in Figure 3A, B).

Case 1 (variant ATTR-CM, 31-year-old, male)

Year	X	X+3	X+10
<u>Electrocardiography</u>			
V1 (mm)	3.0	0.5	0.5
V2 (mm)	4.0	1.0	0.5
V3 (mm)	5.0	2.5	2.5
V1 + V2 +V3 (mm)	12.0	4.0	3.5
<u>Echocardiography</u>			
Septal wall thickness (mm)	10.0	12.0	14.0
Posterior wall thickness (mm)	8.0	11.0	14.0

Case 2 (variant ATTR-CM, 23-year-old, female)

Year	X	X+3	X+10
<u>Electrocardiography</u>			
V1 (mm)	3.0	1.0	0.0
V2 (mm)	6.0	2.0	0.5
V3 (mm)	4.0	3.0	2.0
V1 + V2 +V3 (mm)	13.0	6.0	2.5
<u>Echocardiography</u>			
Septal wall thickness (mm)	9.0	10.0	11.0
Posterior wall thickness (mm)	8.0	9.0	13.0

Case 3 (ATTR-CMwt, 70-year-old, male)

Year	X	X+2	X+8
Electrocardiography			
V1 (mm)	0.5	0.0	0.0
V2 (mm)	2.5	2.5	0.0
V3 (mm)	26.0	16.0	8.0
V1 + V2 +V3 (mm)	29.0	18.5	8.0
Echocardiography			
Septal wall thickness (mm)	8.0	9.0	16.0
Posterior wall thickness (mm)	10.0	10.0	10.0
